# Pilot Study of the Addition of Mass Treatment for Malaria to Existing School-Based Programs to Treat Neglected Tropical Diseases

**DOI:** 10.4269/ajtmh.17-0590

**Published:** 2017-11-06

**Authors:** Lauren M. Cohee, Moses Chilombe, Andrew Ngwira, Samuel K. Jemu, Don P. Mathanga, Miriam K. Laufer

**Affiliations:** 1 Division of Malaria Research, Institute for Global Health, University of Maryland School of Medicine, Baltimore, Maryland;; 2 Malaria Alert Center, University of Malawi College of Medicine, Blantyre, Malawi;; 3 Ministry of Health Malawi, Lilongwe, Malawi

## Abstract

Malaria and neglected tropical diseases (NTDs), including schistosomiasis and soil transmitted helminths, threaten the health of school aged in sub-Saharan Africa. Established school-based mass drug administration (MDA) programs are used to control NTDs. Recent clinical trials have shown benefit of mass treatment of malaria in schools. The potential of adding malaria treatment to existing NTD programs has not been thoroughly evaluated. We offered malaria treatment with artemether-lumefantrine during routine NTD MDA and developed peer education programs in two primary schools in southern Malawi. We assessed participation, safety, and tolerability of coadministration of artemether-lumefantrine with praziquantel and albendazole. Results were compared with two schools conducting standard NTD MDA with additional monitoring by study staff. A total of 3,387 students (68%) received the standard NTD MDA. Among parents who came to schools on the day of the MDA, malaria treatment was well accepted; 87% of students who received the standard NTD MDA in intervention schools also consented for treatment with artemether-lumefantrine. The most frequent treatment emergent adverse events (AEs) were headache and vomiting. However, AEs were rare and were not more frequent in students who received artemether-lumefantrine in addition to praziquantel and albendazole. In this study, we found that the addition of malaria treatment to NTD MDA is well-received and safe. Such integrated programs may leverage existing infrastructures to reduce intervention costs and could become the framework for further integrated school-based health programs.

## INTRODUCTION

In Malawi, as in many other sub-Saharan African countries, school-age children bear a heavy burden of infection and morbidity due to neglected tropical diseases (NTDs) and malaria.^1–4^ NTDs and malaria impact the health of this population leading to anemia and lower educational attainment.^5–10^ A key component of current NTD control policy is at least annual preventive chemotherapy distributed through school-based mass drug administration (MDA).^11,12^ By contrast, current malaria control interventions do not specifically target school-age children despite increasing evidence that school-age children bear the highest burden of infection among all age groups.^13–15^ Clinical trials have shown benefits of school-based mass treatment of malaria, including decreased *Plasmodium falciparum* prevalence, incidence of clinical malaria, and prevalence of anemia.^8,16–21^

Despite the overlap in target population and potential distribution mechanisms, few studies have evaluated the addition of malaria treatment to school-based MDA for NTDs.^19,22,23^ Only one study combined artemisinin-based combination therapy with praziquantel and albendazole treatment, but acceptability and safety outcomes were not reported.^22^ There is no specific contraindication to coadministration of these medications. However, praziquantel is frequently associated with nausea. The combined pill burden of all three medications, especially in older students who require up to nine pills at one time for appropriate dosing of all three drugs, could lead to significant intolerance.

We conducted a pilot study administering artemether-lumefantrine in conjunction with school-based MDA for NTDs and evaluated the safety of the combination. We hypothesized that the intervention would be well tolerated and would increase participation in school-based MDA because of community concern about malaria.

## MATERIALS AND METHODS

### Study design.

The study was conducted at four primary schools in southern Malawi which had previously participated in malaria surveillance studies (unpublished). Two schools were randomly chosen as intervention schools to receive enhanced MDA including peer education program and the addition of antimalarial treatment to the scheduled NTD MDA campaign while two schools served as controls and received routine NTD MDA with additional support and monitoring by study staff.

In all four schools, the routine annual NTD MDA was conducted as recommended by the Ministry of Health. Briefly, school registers are transcribed into an MDA register. Using the register for documentation, an assigned teacher and local Health Surveillance Assistants distribute praziquantel and albendazole over 1 to 2 days with an additional day to treat absent children. Communities are notified about these activities using local networks of information sharing from the health surveillance assistants to village chiefs and community leaders. Consistent with routine procedures, parental consent was not required for NTD MDA participation.

For study purposes, attendance was taken in all schools before the MDA using the school’s MDA register to document who was absent and to identify transfers, dropouts, and students not listed on the register. During the MDA, study staff observed the registration of students and documented the distribution on a study register. At all schools, the health workers and teachers administered praziquantel and albendazole according to standard procedure. Students in intervention and control schools were instructed to come to a study nurse if they were not feeling well and thought they might need medical attention. All adverse events (AEs) were documented. Treatment emergent AEs were defined as undesirable events not present before treatment, or worsening of a preexisting symptom after treatment. AEs were evaluated according to the United States National Institutes of Health Division of AIDS tables for grading the severity of pediatric AEs.^24^ Local health centers were notified of these activities.

Enhanced MDA in the two intervention schools consisted of two components: peer education and the addition of malaria mass treatment to routine school-based NTD MDA. Peer education occurred through health scout clubs, which developed student-led educational presentations about malaria and NTDs to raise awareness among community members about the importance and plan for the MDA activities. Fifteen students per school were chosen by application, teacher endorsements, and audition. Health scouts were trained in the basics of malaria, schistosomiasis and soil transmitted helminth transmission, prevention and treatment as well as community communication techniques by the district Schistosomiasis Coordinator, the district Malaria Coordinator, and a district-level Information, Education and Communication Officer in a 3-day course. Subsequently, health scouts were overseen by two dedicated teachers and a village Health Surveillance Assistant. Health scouts developed songs, poems, and plays to communicate a series of monthly health messages to the community. Health scouts also encouraged participation in the MDA of school age children not enrolled in school. Messages were delivered monthly to students and teachers at school assemblies in junior primary and primary schools, to expectant mothers and younger children at antenatal clinics, and to leaders and other adults in community meetings.

The second part of the intervention consisted of offering mass treatment of malaria with artemether-lumefantrine (Artefan 12/120; Ajanta Pharma Ltd, Mumbai, India) after students participated in the routine NTD MDA. We invited parents to come with their children on the day that their child would receive the routine NTD MDA. Teams of study nurses met with parents and students individually to review inclusion criteria: age 5–15 years and parent or guardian available to provide consent; and exclusion criteria: current illness requiring medical attention, allergy to artemether-lumefantrine, and taken artemether-lumefantrine in the last 7 days. Consent and, if the student was 13 years or older, assent were sought for eligible students. If students were boys or girls less than 12 years of age, they were weighed and the appropriate dose of artemether-lumefantrine was administered by study nurses. Students were given a health passport that included a note about participation in the study. Study staff explained to parents how the remaining doses should be taken. To ensure that artmether-lumefantrine was only given to girls of childbearing age who have infection,^25^ a malaria rapid diagnostic test was performed (Paracheck^®^ Pf; Orchid Biomedical Systems, Goa, India) before treatment of all girls 12 years of age and older. If positive, they were treated as described previously. If negative, they were not treated to avoid any risk of administering artemether-lumefantrine during early pregnancy.

As part of the study in all four schools, children were fed local maize porridge due to concern that too few children would be able to eat before coming to school as is typically encouraged during standard NTD MDA. Because AEs may depend on whether children had eaten, porridge was provided at control and intervention schools.

### Ethical considerations.

The study was reviewed and approved by the University of Malawi College of Medicine Research and Ethics Committee and the Institutional Review Board of the University of Maryland Baltimore. Written informed consent from parents and assent from students 13–15 years of age were obtained in the local language by study staff.

### Statistical analysis.

Data analysis was performed using STATA version 12.1 software (Stata Corp., College Station, TX). Proportions were compared using χ^2^ tests of association. Coverage was defined as the number of students who received either the NTD MDA or artemether-lumefantrine divided by the total number of students on the final MDA registers, including students that were added to the register during attendance taking before and on the days of the NTD MDA. Acceptability was defined as parental support of the intervention demonstrated by attending the routine NTD MDA and providing consent for artemether-lumefantrine administration. With our sample size, we had more than 90% power at alpha 0.05 to detect an increase in the prevalence of AEs from 1% to 2.5%.

## RESULTS

A total of 4,361 students were listed on MDA registers at the four schools before initiation of the study. Attendance surveys before the intervention and on the day of the MDA identified 614 students previously not listed on the school registers. These students were added to the registers yielding a total of 4,976 students on the final registers. Among these, 3,387 students (68%) attended school during the MDA and received the standard NTD treatment.

### Participation in enhanced MDA.

Eighty-seven percent of children who received the standard NTD MDA in intervention schools (1,902/2,193; Table 1) were enrolled in the artemether-lumefantrine study. Fourteen students were excluded because they had received artemether-lumefantrine in the prior 7 days. No students were excluded due to artemether-lumefantrine allergy or illness requiring medical attention. Because girls ≥ 12 years of age were only provided treatment if a malaria rapid diagnostic test was positive, 1,612 students received artemether-lumefantrine (Table 1). Among the parents and students whose parents came to the school on the days of the MDA, there were no refusals. Reasons for parents not coming to the MDA were not assessed.

**Table 1:** Participation in MDA and artemether-lumefantrine distribution

	Control	Intervention
Students on MDA register	1,497	2,864
Students added to the register*	–	–
Enrolled	154 (10%)	275 (10%)
Nonenrolled	10 (1%)	175 (6%)
Final register total	1,661	3,315
Received standard MDA (% final register)	1,194 (72%)	2,193 (66%)
Consented for AL and either treated or RDT tested† (% of those who received MDA)	–	1,902 (87%)
RDT results (girls > 12 yo) # pos/total tested (%)	–	138/428 (32%)

MDA = mass drug administration; AL = artemether-lumefantrine.

* Students identified during attendance surveys and on the day of the MDA who were not listed on the original registers.

† Twenty children were not eligible: eight age > 15 years, 12 currently taking AL.

### Coverage of standard MDA in intervention versus control schools.

Standard MDA coverage was higher in the control schools than in the interventions schools (72% versus 66%, *P* < 0.0001) (Table 1). However, there were more students that were not previously enrolled in school who came to the intervention schools for the MDA. On the day of the MDA, 175 children who were not enrolled in school came for participation in intervention schools compared with only 10 in the control schools (6% versus 1%, *P* < 0.001), suggesting that the intervention increased participation in the standard MDA among children who were not enrolled in school (Table 1). Among students that had dropped out from school since the original school register was made, more attended the enhanced MDA and were treated with praziquantel and albendazole in the intervention schools, but the difference was not statistically significant (20% versus 9%, *P* = 0.2).

### Safety and tolerability of artemether-lumefantrine with praziquantel and albendazole.

We observed 63 AEs in 44 participants because some participants experienced more than one AE. There were 40 AEs in 29 participants that were classified as treatment emergent. Among treatment emergent AEs, 10 (25%) occurred in children who had taken artemether-lumefantrine in addition to praziquantel/albendazole and 30 (75%) occurred in children who had taken only praziquantel/albendazole. The overall AE rate was 1.2% (40/3,387) and was lower in the group that received all three drugs (0.6% versus 1.7%, *P* = 0.004). The most frequent treatment emergent AE was headache (18/40 = 45%) with onset most often 1–8 hours after taking medications (Table 2). However, headache was also the most frequent preexisting, nontreatment emergent condition (14/23 = 61%). Vomiting occurred in three children after taking praziquantel/albendazole and artemether-lumefantrine (3/1,612 = 0.2%) and two children after taking only praziquantel/albendazole (2/1,775 = 0.1%). Both cases of vomiting after only praziquantel/albendazole occurred in children with concomitant illness, including fever. Only one AE was Grade II, all others were Grade I. The grade II event was high fever in a child with 2-day history fever before praziquantel/albendazole treatment who vomited after the medications.

**Table 2:** Treatment emergent adverse events

	Praziquantel albendazole	Artmether-lumefantrine praziquantel albendazole
Number of students who received	1,775	1,612
Headache	15 (0.8%)	3 (0.2%)
Vomiting	2 (0.1%)	3 (0.2%)
Body aches (body pain, muscle aches)	5 (0.3%)	0
Abdominal pain	3 (0.2%)	1 (0.06%)
Fever	2 (0.1%)	2 (0.1%)
Nausea	2 (0.1%)	0
Palpitations	0	1 (0.06%)
Dizziness	1 (0.06%)	0
Total	30 (1.7%)	10 (0.6%)

## DISCUSSION

This study supports the potential for and safety of combining malaria treatment and standard school-based MDA for NTDs. Provision of artemether-lumefantrine following routine praziquantel and albendazole was well-received by parents and was well-tolerated and safe. Although offering malaria treatment did not increase coverage of the standard NTD MDA in the context of a research study, combining malaria treatment with already established platforms for NTD control may increase the cost-effectiveness of both interventions, leading to increased sustainability. Thus, the combination of interventions is an appealing model.

Parents and students in intervention schools were enthusiastic about the opportunity to receive malaria treatment. Almost 90% of parents of children who participated in the standard NTD MDA in intervention schools came to the school, often on more than 1 day of the MDA, to provide consent for malaria treatment. This assessment of participation, obtained in the context of a research study, may either underestimate or overestimate participation an established program. A commonly acknowledged reason research studies may underestimate participation is general anxiety or skepticism about research in some communities. The schools in our study were specifically chosen because of their prior exposure to research and the established trust with our research team. A potentially more substantial reason that our study might underestimate participation is that obtaining informed consent/assent, while necessary, is a significant obstacle to participation in research.^26^ In our study, parents were required to come to the schools to provide consent, which takes time, disrupts the work of the parents and the school days of the students, and, in this setting, the time required for consent was also prolonged because most parents are illiterate. Conversely, research studies may overestimate participation compared with an established program because research participation can be associated with other benefits or incentives. No specific incentive was provided for participants in the intervention schools. However, access to study nurses could have been perceived as an additional benefit. We did not detect concerns related to the acceptability of the intervention but we acknowledge that only interested parents were likely to approach the study team. Ultimately, we hypothesize that participation would likely be increased if combined malaria and NTD MDA were conducted as a program thus not requiring consent. However, more dedicated feasibility and acceptability studies are needed.

It is likely that the time-consuming nature of the consent process also led to decreased overall participation in the intervention schools. Children and their parents were offered enrollment and treatment with artemether-lumefantrine only after praziquantel and albendazole were administered through the standard MDA mechanisms. However, the standard MDA took longer, three full days due the additional procedures of consent and artemether-lumefantrine administration following the standard MDA. In addition, there were delays in the administration of praziquantel and albendazole because there was increased participation of children in the community who were not enrolled in school and therefore required special registration procedures. This was a desired outcome of our study, but may have decreased overall participation at the intervention schools.

When students received artemether-lumefantrine after praziquantel and albendazole, the three-drug combination was not associated with increased AEs. However, all children in both intervention and control schools were provided breakfast consisting of local maize porridge the morning of the MDA. Typically, during MDA for NTDs in Malawi, children are encouraged to eat breakfast at home on the day of the MDA and administration of praziquantel and albendazole may be deferred if students report that they have not eaten. The year of our intervention there was a significant drought in Malawi, leading to a state of emergency with respect to food security. School, Health, and Nutrition coordinators within the Ministry of Health, strongly advised us to provide porridge to the children on the day of the drug administration due to the likelihood that many students would not have food available at home. Thus, the three-drug combination may be less well tolerated if children take medicines on an empty stomach. Based on age-specific risks of malaria and NTDs within the primary school age range, it may be possible to target treatments to different age groups alleviating the need to administer all drugs together but maintain the benefits of combined delivery mechanisms.

Adding malaria treatment to MDA for NTDs has the potential to improve the cost-benefit ratio of both interventions.^27,28^ However, for malaria treatment in schools to successfully improve health, treatment is likely required more frequently than the annual or biannual NTD MDAs and in some settings at specific times based on the seasonality of malaria transmission. In settings with highly seasonal malaria transmission, treatment at the end of the transmission season leads to prolonged parasite free time making the timing of the distribution critical.^21^ In Malawi, annual NTD MDA occurs in April, which is also the end of the malaria high transmission season. However, on-going transmission throughout the year may make this timing less critical than the frequency of distributions. Programs to deliver additional malaria treatments could be developed independently or other school-based health interventions, such as administration of the human papilloma virus vaccine, could be paired with other malaria control visits. Ultimately such integration could be the platform for a school-based health system, which could provide a variety of health services to children who would typically not have routine interaction with the health care system from when they complete their primary immunizations until, for girls, enrollment in antenatal care.

Overall our results support consideration of combining malaria treatment with school-based MDA to control schistosomiasis and soil transmitted helminths.
